# Glioma Migration Through the Corpus Callosum and the Brainstem Detected by Diffusion and Magnetic Resonance Imaging: Initial Findings

**DOI:** 10.3389/fnhum.2019.00472

**Published:** 2020-02-25

**Authors:** Guive Sharifi, Amir Mohammad Pajavand, Saeedeh Nateghinia, Tohid Emami Meybodi, Hossein Hasooni

**Affiliations:** ^1^Skull Base Research Center, Loghman Hakim Hospital, Shahid Beheshti University of Medical Sciences, Tehran, Iran; ^2^Loghman Hakim Hospital, Shahid Beheshti University of Medical Sciences, Tehran, Iran; ^3^Institute for Cognitive and Brain Sciences, Shahid Beheshti University GC, Tehran, Iran; ^4^Cellular and Molecular Research Center, Iran University of Medical Sciences, Tehran, Iran

**Keywords:** tract-based spatial statistics, voxel-based morphometry, magnetic resonance imaging, corpus callosum, white matter

## Abstract

**Purpose**: Glioma cell infiltration, in which the glioma tumor cells spread long distances from the primary location using white matter (WM) or blood vessels, is known as a significant challenge for surgery or localized chemotherapy and radiation therapy. Following the World Health Organization (WHO), the glioma grading system ranges from stages I to IV, in which lower-grade gliomas represent benign tumors, and higher grade gliomas are considered the most malignant.

**Materials and Methods**: We gathered magnetic resonance imaging (MRI) and diffusion tensor imaging (DTI) data for seven patients with right precentral gyrus-located tumors and six age- and sex-matched healthy subjects for analysis. Tract-Based Spatial Statistics (TBSS) was utilized to evaluate whole-brain WM implication due to probable tumor infiltration. Also, along-tract statistics were used in order to trace the implicated WM tracts. Finally, for cortical evaluation of probable tumor cell migration, voxel-based morphometry (VBM) was utilized, which allowed us to do whole-brain cortical estimation.

**Results**: The TBSS results revealed significantly higher fractional anisotropy (FA) and lower mean diffusivity (MD) in the left side superior corona radiata. Also, higher FA was observed in the right corticostriatal tract. Along-tract statistics were also compiled on the corpus callosum (CC), which is anatomically known as a hub between hemispheres. The body of the CC, which connected with the superior corona radiata anatomically, showed significantly higher FA values relative to healthy subjects, which are in line with the TBSS results. Consistent with these results, whole-brain gray matter changes were analyzed *via* VBM, which showed significant hypertrophy of both sides of the brainstem.

**Conclusion**: In future investigations, focusing on the genetic basis of the glioma patients in line with imaging studies on a larger sample size, which is known as genetics imaging, would be a suitable approach for tracing this process.

## Introduction

Glioma invasion, as the major obstacle for curing patients, is hypothesized to be carried out *via* extracellular routes to the other brain structures, and causes significant complications for complete surgical resection and chemotherapy and radiation therapy.

Glioma cell migration, which can occur over long distances *via* white matter (WM) or blood vessels to injure WM or cortical structures, has been investigated previously in mammalian brains (Cayre et al., 2009). Scherer (1938) investigated 100 patients with glioma tumors and created criteria for glioma invasion through the brain parenchyma, preexisting blood vessels, subarachnoid space, and WM tracts.

Diffusion tensor imaging (DTI) is a modern technique that is sensitive to the diffusion of water flow along the WM tracts. Hence, it can detect tumor infiltration through the WM tracts when conventional magnetic resonance imaging (MRI) scans appear to be normal (Price et al., 2003).

The infiltrating glioma cells extend beyond a surgeon’s reach, which can limit the effectiveness of localized therapy (Hochberg and Pruitt, 1980; Burger et al., 1988; Kreth et al., 1993; Shapiro, 1999).

Regarding the symptoms of right precentral gyrus glioma patients, sensory deficits in the contralateral side are usually represented in these patients, which could be due to atrophy or even hypertrophy of sensory cortices, such as the primary and secondary somatosensory cortex (SSC), the brainstem, and insula.

**Figure 1:**
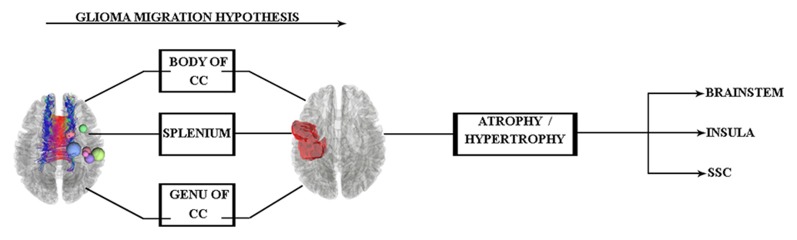
Glioma tumor migration hypothesis between hemispheres and its probable effect on cortical regions of the contralateral side. Abbreviations: CC, corpus callosum; SSC, somatosensory cortex.

The pathway of glial cell migration from the left precentral gyrus to its contralateral side and its effects on cortical regions are the main focus of this study. Therefore, according to the aforementioned findings, we hypothesized that (see Figure 1) the glial cells of left precentral gyrus tumors may migrate along the corpus callosum (CC) WM tract, which acts as a hub between hemispheres. The specific region of the CC involved is not yet known and needs to be clarified in the study.

Next, if glioma migration is proved by the results, the probable cortical changes of the left hemisphere also need to be investigated. That is why, we used DTI techniques [deterministic tractography, tract-based spatial statistics (TBSS), and along-tract statistics] to clarify this probable mechanism.

## Materials and Methods

### Participants

In this study, seven patients with different types of glioma tumors of the right motor cortex (see Table 1; Figure 1) and seven healthy subjects (age- and sex-matched) were included. Subjects with previous neurological problems, such as Parkinson’s disease or previous cancer history, were excluded. In addition, in order to prevent statistical problems, we performed a two-tailed *t*-test, and no significant differences were seen in either the sex (*P* = 0.2) or age (*P* = 0.32) of the groups.

**Table 1:** Demographic and clinical data of the study population.

	Patients	Controls	*T*-value	*P*-value
	7	6	-	-
Age	45 ± 11	45 ± 12	0.30	0.76*
Sex (Male)	5 (2)	4 (2)	-	-
**Pathology results**
1. Diffuse astrocytoma with foci of anaplastic transformation (grade II)
2. Oligodendroglioma, WHO grade II
3. Oligodendroglioma, WHO grade II
4. Gemistocytic astrocytoma with anaplastic transformation, WHO grade III
5. Ganglioglioma, WHO grade I
6. Ganglioglioma, WHO grade I
7. Oligoastrocytoma WHO grade II				

Seven patients with no cancer background or metastatic tumor were included in the study. Values are expressed as mean ± SD. *P-value calculated independent two-sample t-test.

### Image Acquisition

#### Structural MRI and DTI Data Acquisition

All structural MRI scans were acquired from 3T MRI scanners (Siemens Prisma). 3D T1 MPRAGE anatomic acquisitions was done (1 mm slices, 256 × 256 matrix, echo time [TE] = 3.74 ms, repetition time [TR] = 1810 ms, flip angle = 30°) and used to superimpose DTI images.

Also, diffusion-weighted imaging (DWI) brain scan was done *via* the same scanner with a 64-channel head coil. Other acquisition parameters were as follows: number of slices, 68; diffusion directions, 30; FOV, 256 × 256 mm^2^; voxel size, 2 × 2 × 2 mm^3^; TR/TE, 9,000/90 ms.

### Data Analysis

#### Structural Data Preprocessing and Voxel-Based Morphometry (VBM)

Structural data were analyzed using FSL-VBM protocol (Ashburner and Friston, 2000; Good et al., 2001). First, nonbrain tissues were extracted using the brain extracting tool (BET; Smith, 2002) and GM-segmented before MNI152 standard space registration using nonlinear registration.

**Figure 2:**
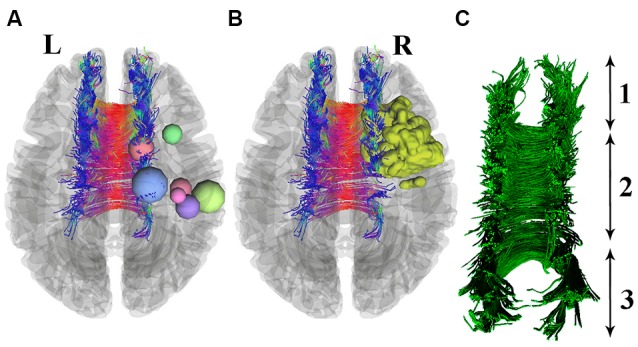
Seven patients with right precentral gyrus glioma (nodes are used for better visualization) are shown in section **(A)**. One patient with a 3D-segmented tumor is visualized as an example in part **(B)**. Deterministic tractography of CC is presented in part **(C)**, which includes its whole neuroanatomy: (1) Genu of CC; (2) Body of CC; and (3) Splenium.

The resulting GM partial volume images were averaged to create a study template, and then all native GM images were registered nonlinearly to this template. The modulated segmented images were then smoothed with an isotropic Gaussian kernel with a sigma of 3 mm. For the last step, the voxel-wise general linear model (GLM) was applied using permutation-based testing. Threshold-free cluster enhancing (TFCE) was used for thresholding (Smith and Nichols, 2009). Images were thresholded at *P* < 0.05 and corrected for multiple comparisons (see Figure 2).

#### DTI Data Preprocessing and TBSS

In order to preprocess the DTI data, we utilized the FMRIB Software Library (FSL 5)^1^. At first, all diffusion-weighted images were checked visually for any visible artifacts and then corrected for B_0_ inhomogeneities and eddy-current distortion, with each subject’s DWI registered to the corresponding *b* = 0 images *via* affine transformation.

Second, the data were brain-extracted *via* FSL BET to remove unwanted voxels, and finally, fractional anisotropy (FA), mean diffusivity (MD), axial diffusivity (AD), and eigenvector L1, L2, and L3 maps were created using DTIFIT.

For analyzing WM changes between groups, TBSS was performed (Smith et al., 2006) using Functional Magnetic Resonance Imaging of the Brain (FMRIB) Software Library (FSL) version 5.0 (Smith et al., 2004). For the first step, all FA images were nonlinearly aligned to a common space (FMRIB58FA_1 mm). In order to create a mean FA skeleton, mean FA images were created for each subject, and each FA image was projected onto the mean FA skeleton. TBSS was also performed for non-FA data (MD and RD). Nonlinear registration was obtained for FA data, and each MD image was projected to the mean FA skeleton.

### Statistical Analyses

#### Voxel and Tract-Based Statistics

A voxel-wise GLM was performed for structural T1 data after performing the VBM protocol using 5,000 permutations; finally, TFCE was used for finding significant clusters with thresholds set at *p* < 0.05, corrected for multiple comparisons (Smith and Nichols, 2009).

Statistical analyses for DTI data were performed using voxel-wise statistical analysis of FA, MD data using TBSS V1.2 part of FSL (Smith et al., 2006), and FA, MD changes were assessed using permutation-based nonparametric testing with 5,000 random permutations (Nichols and Holmes, 2002). Our statistical threshold was TFCE and the family-wise error-corrected *P*-value of 0.05 (Smith and Nichols, 2009).

After performing voxel-wise statistics, MNI coordinates of significant clusters in GM were calculated, and anatomical regions were identified using Harvard subcortical and Juelich atlases.

#### Along-Tract Statistics

VBM and TBSS allowed us to evaluate the cortical and WM alterations in glioma patients relative to healthy subjects, but in order to achieve the disclosure of glioma invasion patterns along WM tracts, we used along-tract statistical analyses (Colby et al., 2012) to show the probable hub of this invasion.

Based on this technique, which could be a suitable complement for TBSS, the FA values along the significant tracts that were visualized by TBSS were extracted again separately to determine the WM hypertrophy or demyelination probability in glioma patients vs. healthy controls.

According to the small sample size, we decided to use a linear mixed-effects model, in which the FA is the only dependent variable. Hence, using the along-tract statistics MATLAB toolbox^2^ allowed us to extract FA values and streamlines, and the standard deviation of the WM tract was added to the other variables for application on WM tract data as a serial univariate approach. Then fixed-effect results containing the position factor, overall intercept, and group:position interaction were analyzed.

All the procedures of statistical analyses were performed *via* R version 3.5.0 (R Core Team, 2018) and MATLAB (MathWorks, Natick, MA, USA) software.

## Results

### Tract-Based Spatial Statistics

**Figure 3:**
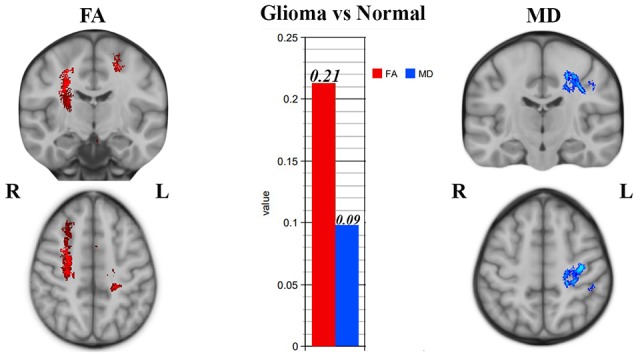
Tract-based spatial statistics (TBSS) results showing significant superior corona radiata and corticostriatal tract alterations in patients vs. normal subjects. Significantly higher fractional anisotropy (FA) and lower mean diffusivity (MD) values were seen in CC, which could be a sign of white matter (WM) maturation.

Significant WM maturation occurred in patients vs. healthy controls, and the FA was significantly higher (*P* < 0.001) than the MD in patients relative to healthy subjects (see Figure 3); this result could be a reflection of probable WM maturation due to glioma tumor migration.

According to Figure 3, right corticostriatal pathways in the ipsilateral side of the tumor’s location showed higher FA but no significant MD changes. Hence, we cannot judge whether there was WM maturation or demyelination.

According to Figure 3, the left superior corona radiata showed WM maturation as indicated by the DTI metrics. The FA was significantly higher than the MD values (*P* < 0.001), which could be a sign of glioma migration along the CC, which acts as a hub between both hemispheres.

In keeping with our hypothesis diagram (see Figure 1), glioma migration into the contralateral side of the tumors’ location is observed, but consistent with TBSS limitations (Bach et al., 2014), specific disclosure of glioma migration along the WM route would not be possible. Hence, we performed along-tract statistics to clarify whether there was a matured WM hub between hemispheres.

### Along-Tract Statistics

In order to verify our probable WM pattern prediction of glioma migration, FA values were analyzed along the tract of the CC, which is anatomically known as a hub between hemispheres.

**Figure 4:**
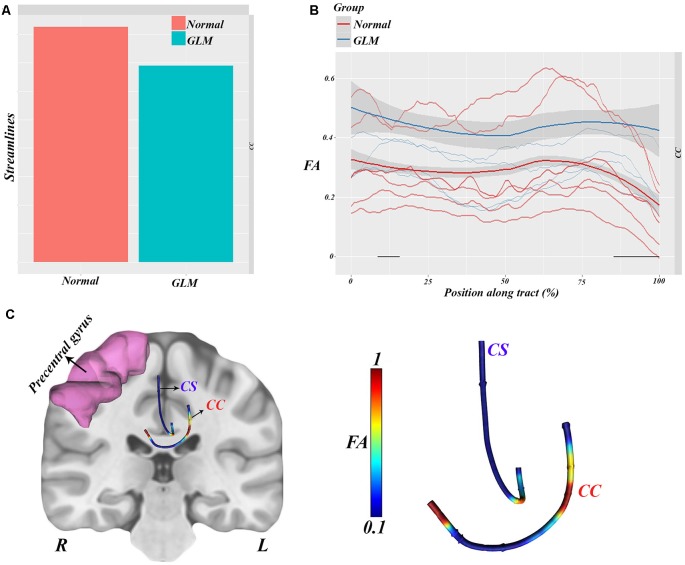
Along-tract statistical analyses showing significantly lower streamlines in glioma patients relative to normal subjects (**A**). FA values along CC were analyzed between groups, which showed a remarkable difference (higher FA along streamlines) in patients vs. controls (**B**). All the statistical features visualized show the CC implication as a hub between hemispheres (**C**).

As, we show in Figure 4A, streamlines between groups were analyzed, and lower streamlines but no signs were found in glioma patients vs. healthy subjects (*t* = −1.87, *p* = 0.065). According to the visualized results in Figures 4B,C, along-tract statistical analyses were carried out in order to reveal the overall FA vs. position curves between the groups.

This analysis showed significant group–position implication (*F* = 33.3, *p* = 0.00018) in the body of the CC and right corticostriatal WM, which are illustrated in Figure 4C.

Hence, all the statistical results can be visualized in Figure 4C, which is consistent with the TBSS results showing higher FA in the left superior corona radiata in glioma patients.

**Figure 5:**
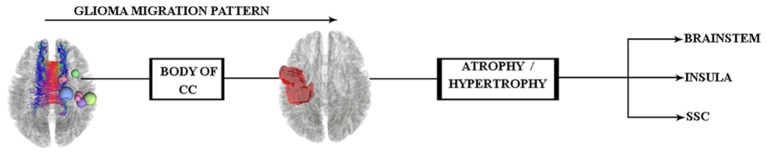
Hypothesis of glioma migration from the right precentral gyrus to the contralateral side updated by the along-tract statistics results, which reveal this transition through the body of the CC.

According to these results and our hypothesis diagram (see Figure 5), the body of the CC played a significant role in glioma migration from the precentral gyrus to the contralateral side (see Figure 4). This migration would induce cortical changes, such as atrophy or hypertrophy of regions, which need to be verified by cortical imaging techniques, as we have done in this study.

### Voxel-Based Morphometry

**Figure 6:**
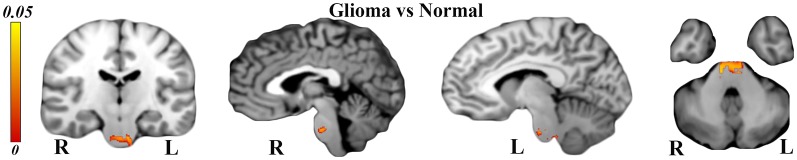
Voxel-based morphometry (VBM) analyses showing significantly hypertrophied brainstem on both sides [*P* < 0.05 and threshold-free cluster enhancing (TFCE) corrected] in glioma patients relative to normal subjects.

Structural T1 analyses *via* VBM revealed brainstem hypertrophy on both sides (see Figure 6) in glioma patients in comparison to healthy controls (TFCE corrected, *P*-value < 0.05). Our analyses also showed atrophied Brodmann areas 4, 6, and 31, but this was not significant (*P* > 0.05).

As indicated by the TBSS and along-tract statistics results, glioma migration through the body of the CC involved the left side precentral gyrus and also increased the FA values of the corticostriatal tracts, which could be the reason for the right brainstem hypertrophy.

However, WM analyses of the corticospinal tracts or internal capsule gave no significant results to justify the probable reason for the left side hypertrophy of the brainstem.

**Figure 7:**
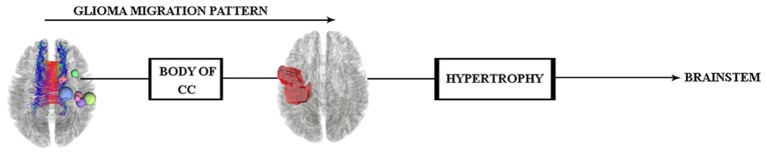
Glioma migration hypothesis, which shows the transition pattern along the body of the CC and probable influence (hypertrophy) on both sides of the brainstem.

Altogether, all the results investigated by WM and gray matter analyses would indicate that the brainstem hypertrophy occurred because of the glioma migration (see Figure 7), and in order to trace this transition *in vivo*, future longitudinal investigations are needed.

## Discussion

### Present Study

In the present study, we had seven patients with right precentral gyrus glioma, and the pathological examinations verified the tumor type. Following our hypothesis diagram (see Figure 1) and in keeping with previous literature, we predicted glioma migration through the CC from the left side precentral gyrus into its contralateral side. Subsequently, this probable migration would change in cortical regions, which needs evaluation.

In order to evaluate cortical changes due to glioma migration, we used VBM analyses, which allowed us to determine the whole-brain cortical volume. Interestingly, both sides of the brainstem showed hypertrophy, which could be a reflection of glioma cell aggregation in this region.

TBSS analyses were performed for the prediction of glioma migration within whole-brain WM tracts, and the results supported our hypothesis of probable glioma migration along the CC to the right-side superior corona radiata. The involvement of WM tracts in glioma tumor cell migration has been shown to be a significant process (Pedersen et al., 1995; Bjerkvig et al., 1997; Soroceanu et al., 1999), which is in line with our TBSS results on superior corona radiata of the left side hemisphere with WM fiber maturation.

The involvement of the CC, which is known as a Scherer’s structure in this process (Scherer, 1938), needs investigation *via* a complementary analysis to TBSS, which is carried out by along-tract statistics. The results showed maturation of the body of the CC, which is in line with TBSS results that showed no significant WM demyelination signs.

As stated in our study’s hypothesis (see Figures 1, 5), probable cortical changes would occur as a consequence of glioma migration from the right precentral gyrus to its contralateral side, but to the best of our knowledge, no previous glioma cell invasion to the brainstem due to this isolated process has been reported.

### White Matter Tract Maturation in the Glioma Migration Process

Consistent with the WM analysis results, WM maturation was seen in the right corticostriatal pathways, left superior corona radiata, and the body of the CC, which showed significantly increased FA and decreased MD values. The lack of demyelination signs in WM tracts would be a reflection of glioma cell migration along WM tracts to the contralateral side of the tumor’s location, rather than degeneration.

However, we excluded the patients with previous metastatic tumor history or high-grade gliomas, and our WM analyses are in line with a study that reported higher MD and lowered FA in the peritumoral region of metastatic tumor patients (Holly et al., 2018). Interestingly, higher FA and lower MD values were observed in the ipsilateral peritumoral regions of glioma patients.

A few DTI studies on high-grade glioma patients have also claimed to show signs of glioma cell migration through the CC into the contralateral side (Price et al., 2004). In an investigation on 31 high-grade glioma patients utilizing DTI, the tumor-related area of the CC showed reduced FA and increased ADC values (Kallenberg et al., 2013).

These changes in anisotropy and diffusivity are in line with our results, which suggest tumor spread along the CC, but exclusion of high-grade glioma patients and analysis of the CC *via* along-tract statistics for increasing the accuracy are the novel aspects of our study.

In contrast to our study, in an investigation of glioma infiltrating from edema tissue, no significant difference was seen in FA and MD. However, the study population contained 18 glioblastomas and 22 metastatic tumors, which could be a limitation for this study (Hoefnagels et al., 2014). In addition, the DTI study was carried out between the tumoral and peritumoral site and the contralateral side, which would be another significantly different approach from what we claimed in this study.

### Hypertrophy of Brainstem and Glioma Migration

The propagation of tumor cells throughout the whole brain and its overall invasion pattern is not yet clear, and more studies are needed for investigation in this context.

In accordance with the related symptoms of precentral gliomas, the involvement of the cortical homunculus (Penfield and Boldrey, 1937) is expected, and obviously, the main sensory projections from the brainstem into the cortical homunculus are also prominent. In keeping with these considerations, gray matter analyses in our study showed significant hypertrophy of the brainstem in glioma patients relative to healthy subjects.

This result may be a reflection of the tumor cell migration, which is in line with a histopathological investigation on mouse models that reported the accumulation of tumor cells in regions far from the transplant side (Mughal et al., 2018).

However, with the dispersal of glioma cells through the mouse brains, surprisingly, the hippocampus was remarkably free of glioma cell infiltration, which may be in line with our cortical analyses *via* VBM that showed no involvement of both sides of the hippocampus in this process.

Knowledge of tumor cell aggregation in located regions of the brain is also important in radiotherapy, so that the structures with accumulated tumor cells could receive higher dosage radiation, and the tumor-free structures could be relatively spared (Nourallah et al., 2017).

However, glioma invasion has been speculated to occur by invasion along vessels in the perivascular spaces (Cuddapah et al., 2014), but the present study and our results may not support this hypothesis. Nevertheless, more investigation with different approaches is needed to elucidate this process.

### Precision Medicine

Precision medicine is currently known as a treatment paradigm considering the molecular and cellular features of a tumor along with its supplementary properties, such as genetics, in order to create a tailor-made treatment (Yates et al., 2018).

According to the World Health Organization (WHO), the glioma grading system ranges from stages I to IV, in which the lower grade (I–II) gliomas are considered to be benign tumors, and the higher-grade gliomas (III–IV) are considered malignant (Louis et al., 2007; Barchana et al., 2012; Ostrom et al., 2014).

However, there is no distinct genetic profile for benign or malignant gliomas to promote tailored therapies for these tumors, so producing new information about glioma cell properties such as migration, behavior, and genetics would be a crucial step toward the realization of precision medicine.

These considerations along with the modern developed multidisciplinary investigation methods, such as genetic imaging, would be a suitable approach to complement other cellular, molecular, and genetic approaches (Mackey et al., 2016).

Large-scale distributed analyses of MRI scans combined with a voxel-wise genome-wide association approach would be a useful tool in the future to prescribe tailored therapy for gliomas. However, these approaches are currently being adopted in the study of neurological disorders, and the results are quite promising (Thompson et al., 2010; Jahanshad et al., 2018).

Altogether, in gathering glioma genetic, cellular, and behavior imaging in order to reach a purpose-based criterion for future glioma precision therapy, taking advantage of previous investigations on neurological disorders using the same approach is obviously needed (Hampel et al., 2017, 2018; Titova and Chaudhuri, 2017; Ferretti et al., 2019).

## Conclusion

In the present study, the glioma migration hypothesis was evaluated using DTI and structural MRI analyses. Based on the tumor location (precentral gyrus), the body of the CC showed significant alterations according to the DTI analyses. In addition, both sides of the brainstem also showed hypertrophy, which could be a reflection of glioma cell aggregation far from the primary tumor area.

It should be mentioned that the sample size of the present study is a major limitation, and future investigations need to overcome this shortcoming. Genome-wide association analyses in keeping with multimodal imaging analyses, such as genetic imaging, would be a practical approach to moving forward in precision tailored therapy.

## Data Availability Statement

The datasets analyzed in this article are not publicly available. Requests to access the datasets should be directed to shahryarpajavand@outlook.com.

## Ethics Statement

The studies involving human participants were reviewed and approved by Skull Base Research Center, Loghman Hakim Hospital, Shahid Beheshti University of Medical Sciences, Tehran, Iran. Written informed consent for participation was not required for this study in accordance with the national legislation and the institutional requirements.

## Author Contributions

AP acquired, analyzed, and explained the data, drafted the manuscript and revised it. GS is the main neurosurgeon of the cases. AP, HH, SN, and TM revised the manuscript.

## Conflict of Interest

The authors declare that the research was conducted in the absence of any commercial or financial relationships that could be construed as a potential conflict of interest.

## Footnotes

^1^http://www.fmrib.ox.ac.uk/fsl

^2^http://github.com/johncolby/along-tract-stats

## References

[B1] Ashburner J. Friston K. J. (2000). Voxel-based morphometry—the methods. Neuroimage 11, 805–821. 10.1006/nimg.2000.0582 10860804

[B2] Bach M. Laun F. B. Leemans A. Tax C. M. Biessels G. J. Stieltjes B.. (2014). Methodological considerations on tract-based spatial statistics (TBSS). Neuroimage 100, 358–369. 10.1016/j.neuroimage.2014.06.021 24945661

[B3] Barchana M. Margaliot M. Liphshitz I. (2012). Changes in brain glioma incidence and laterality correlates with use of mobile phones—a nationwide population based study in Israel. Asian Pac. J. Cancer Prev. 13, 5857–5863. 10.7314/apjcp.2012.13.11.5857 23317269

[B4] Bjerkvig R. Lund-Johansen M. Edvardsen K. (1997). Tumor cell invasion and angiogenesis in the central nervous system. Curr. Opin. Oncol. 9, 223–229. 10.1097/00001622-199709030-00002 9229143

[B5] Burger P. C. Heinz E. R. Shibata T. Kleihues P. (1988). Topographic anatomy and CT correlations in the untreated glioblastoma multiforme. J. Neurosurg. 68, 698–704. 10.3171/jns.1988.68.5.0698 2833587

[B6] Cayre M. Canoll P. Goldman J. E. (2009). Cell migration in the normal and pathological postnatal mammalian brain. Prog. Neurobiol. 88, 41–63. 10.1016/j.pneurobio.2009.02.001 19428961 PMC2728466

[B7] Colby J. B. Soderberg L. Lebel C. Dinov I. D. Thompson P. M. Sowell E. R. (2012). Along-tract statistics allow for enhanced tractography analysis. NeuroImage 59, 3227–3242. 10.1016/j.neuroimage.2011.11.004 22094644 PMC3288584

[B8] Cuddapah V. A. Robel S. Watkins S. Sontheimer H. (2014). A neurocentric perspective on glioma invasion. Nat. Rev. Neurosci. 15, 455–465. 10.1038/nrn3765 24946761 PMC5304245

[B9] Ferretti M. T. Santuccione-Chadha A. Hampel H. (2019). Account for sex in brain research for precision medicine. Nature 569:40. 10.1038/d41586-019-01366-5 31040416

[B10] Good C. D. Johnsrude I. S. Ashburner J. Henson R. N. Friston K. J. Frackowiak R. S. (2001). A voxel-based morphometric study of ageing in 465 normal adult human brains. NeuroImage 14, 21–36. 10.1006/nimg.2001.0786 11525331

[B12] Hampel H. O’Bryant S. E. Durrleman S. Younesi E. Rojkova K. Escott-Price V.. (2017). A precision medicine initiative for Alzheimer’s disease: the road ahead to biomarker-guided integrative disease modeling. Climacteric 20, 107–118. 10.1080/13697137.2017.1287866 28286989

[B11] Hampel H. Vergallo A. Giorgi F. S. Kim S. H. Depypere H. Graziani M.. (2018). Precision medicine and drug development in Alzheimer’s disease: the importance of sexual dimorphism and patient stratification. Front. Neuroendocrinol. 50, 31–51. 10.1016/j.yfrne.2018.06.001 29902481

[B13] Hochberg F. H. Pruitt A. (1980). Assumptions in the radiotherapy of glioblastoma. Neurology 30, 907–911. 10.1212/wnl.30.9.907 6252514

[B14] Hoefnagels F. W. De Witt Hamer P. Sanz-Arigita E. Idema S. Kuijer J. P. Pouwels P. J.. (2014). Differentiation of edema and glioma infiltration: proposal of a DTI-based probability map. J. Neurooncol. 120, 187–198. 10.1007/s11060-014-1544-9 25079117

[B15] Holly K. S. Fitz-Gerald J. S. Barker B. J. Murcia D. Daggett R. Ledbetter C.. (2018). Differentiation of high-grade glioma and intracranial metastasis using volumetric diffusion tensor imaging tractography. World Neurosurg. 120, e131–e141. 10.1016/j.wneu.2018.07.230 30165214

[B16] Jahanshad N. Roshchupkin G. Faskowitz J. Hibar D. P. Gutman B. A. Adams H. H. H. (2018). “Chapter one—multisite metaanalysis of image-wide genome-wide associations with morphometry,” in Imaging Genetics, eds Dalca A. V. Batmanghelich N. K. Shen L. Sabuncu M. R. (Academic Press), 1–23.

[B17] Kallenberg K. Goldmann T. Menke J. Strik H. Bock H. C. Stockhammer F.. (2013). Glioma infiltration of the corpus callosum: early signs detected by DTI. J. Neurooncol. 112, 217–222. 10.1007/s11060-013-1049-y 23344787 PMC3607728

[B18] Kreth F. W. Warnke P. C. Scheremet R. Ostertag C. B. (1993). Surgical resection and radiation therapy versus biopsy and radiation therapy in the treatment of glioblastoma multiforme. J. Neurosurg. 78, 762–766. 10.3171/jns.1993.78.5.0762 8385709

[B19] Louis D. N. Ohgaki H. Wiestler O. D. Cavenee W. K. Burger P. C. Jouvet A.. (2007). The 2007 WHO classification of tumours of the central nervous system. Acta Neuropathol. 114, 97–109. 10.1007/s00401-007-0243-4 17618441 PMC1929165

[B20] Mackey S. Kan K. J. Chaarani B. Alia-Klein N. Batalla A. Brooks S.. (2016). Genetic imaging consortium for addiction medicine: from neuroimaging to genes. Prog. Brain Res. 224, 203–223. 10.1016/bs.pbr.2015.07.026 26822360 PMC4820288

[B21] Mughal A. A. Zhang L. Fayzullin A. Server A. Li Y. Wu Y.. (2018). Patterns of invasive growth in malignant gliomas-the hippocampus emerges as an invasion-spared brain region. Neoplasia 20, 643–656. 10.1016/j.neo.2018.04.001 29793116 PMC6030235

[B22] Nichols T. E. Holmes A. P. (2002). Nonparametric permutation tests for functional neuroimaging: a primer with examples. Hum. Brain Mapp. 15, 1–25. 10.1002/hbm.1058 11747097 PMC6871862

[B23] Nourallah B. Digpal R. Jena R. Watts C. (2017). Irradiating the subventricular zone in glioblastoma patients: is there a case for a clinical trial? Clin. Oncol. 29, 26–33. 10.1016/j.clon.2016.09.005 27729188

[B24] Ostrom Q. T. Gittleman H. Liao P. Rouse C. Chen Y. Dowling J.. (2014). CBTRUS statistical report: primary brain and central nervous system tumors diagnosed in the United States in 2007–2011. Neuro Oncol. 16, iv1–iv63. 10.1093/neuonc/nou223 25304271 PMC4193675

[B25] Pedersen P. H. Edvardsen K. Garcia-Cabrera I. Mahesparan R. Thorsen J. Mathisen B.. (1995). Migratory patterns of lac-z transfected human glioma cells in the rat brain. Int. J. Cancer 62, 767–771. 10.1002/ijc.2910620620 7558428

[B26] Penfield W. Boldrey E. (1937). Somatic motor and sensory representation in the cerebral cortex of man as studied by electrical stimulation. Brain 60, 389–440. 10.1093/brain/60.4.389

[B28] Price S. J. Burnet N. G. Donovan T. Green H. A. Peña A. Antoun N. M.. (2003). Diffusion tensor imaging of brain tumours at 3T: a potential tool for assessing white matter tract invasion? Clin. Radiol. 58, 455–462. 10.1016/s0009-9260(03)00115-6 12788314

[B27] Price S. J. Peña A. Burnet N. G. Pickard J. D. Gillard J. H. (2004). Detecting glioma invasion of the corpus callosum using diffusion tensor imaging. Br. J. Neurosurg. 18, 391–395. 10.1080/02688690400005255 15702843

[B29] Scherer H. J. (1938). Structural development in gliomas. Am. J. Cancer 34, 333–351.

[B31] Shapiro W. R. (1999). Current therapy for brain tumors: back to the future. JAMA Neurol. 56, 429–432. 10.1001/archneur.56.4.429 10199330

[B32] Smith S. M. (2002). Fast robust automated brain extraction. Hum. Brain Mapp. 17, 143–155. 10.1002/hbm.10062 12391568 PMC6871816

[B35] Smith S. M. Jenkinson M. Johansen-Berg H. Rueckert D. Nichols T. E. Mackay C. E.. (2006). Tract-based spatial statistics: voxelwise analysis of multi-subject diffusion data. NeuroImage 31, 1487–1505. 10.1016/j.neuroimage.2006.02.024 16624579

[B34] Smith S. M. Jenkinson M. Woolrich M. W. Beckmann C. F. Behrens T. E. Johansen-Berg H.. (2004). Advances in functional and structural MR image analysis and implementation as FSL. Neuroimage 23, S208–S219. 10.1016/j.neuroimage.2004.07.051 15501092

[B33] Smith S. M. Nichols T. E. (2009). Threshold-free cluster enhancement: addressing problems of smoothing, threshold dependence and localisation in cluster inference. NeuroImage 44, 83–98. 10.1016/j.neuroimage.2008.03.061 18501637

[B36] Soroceanu L. Manning T. J. Jr. Sontheimer H. (1999). Modulation of glioma cell migration and invasion using Cl^−^ and K^+^ ion channel blockers. J. Neurosci. 19, 5942–5954. 10.1523/JNEUROSCI.19-14-05942.1999 10407033 PMC6783071

[B37] Thompson P. M. Martin N. G. Wright M. J. (2010). Imaging genomics. Curr. Opin. Neurol. 23, 368–373. 10.1097/WCO.0b013e32833b764c 20581684 PMC2927195

[B38] Titova N. Chaudhuri K. R. (2017). Personalized medicine in Parkinson’s disease: time to be precise. Mov. Disord. 32, 1147–1154. 10.1002/mds.27027 28605054 PMC5575483

[B39] Yates L. R. Seoane J. Le Tourneau C. Siu L. L. Marais R. Michiels S.. (2018). The european society for medical oncology (ESMO) precision medicine glossary. Ann. Oncol. 29, 30–35. 10.1093/annonc/mdx707 29140430

